# Factors Associated with Fatality during the Intensive Phase of Anti-Tuberculosis Treatment

**DOI:** 10.1371/journal.pone.0159925

**Published:** 2016-08-03

**Authors:** T. Rodrigo, M. Casals, J. A. Caminero, J. M. García-García, M. A. Jiménez-Fuentes, J. F. Medina, J. P. Millet, J. Ruiz-Manzano, J. Caylá, Working Group of the Integrated Programme of Tuberculosis Research

**Affiliations:** 1 Programa Integrado de Investigación en Tuberculosis (PII TB), Fundación Respira de la Sociedad Española de Neumología y Cirugía Torácica (SEPAR), Barcelona, Spain; 2 Centro de Investigación Biomédica en Red de Epidemiología y Salud Pública (CIBERESP), Instituto de Salud Carlos III, Ministerio de Economía y Competitividad, Madrid, Spain; 3 Servicio de Neumología. Hospital General Universitario de Gran Canaria Dr, Negrín, Canary Islands, Spain; 4 Servicio de Neumología, Hospital San Agustín de Avilés, Asturias, Spain; 5 Unidad de Tuberculosis, Hospital Universitario Valle de Hebrón, Barcelona, Spain; 6 Servicio de Neumología, Hospital Universitario Virgen del Rocío, Sevilla, Spain; 7 Servicio de Neumología, Hospital Universitario Germans Trías y Pujol de Badalona, Badalona, Spain; 8 Centro de Investigación Biomédica en Red de Enfermedades Respiratorias (CIBERES), Instituto de Salud Carlos III, Ministerio de Economía y Competitividad, Madrid, Spain; 9 International Union Against Tuberculosis and Lung Disease, París, France; 10 Unidad de Investigación de Tuberculosis, Servicio de Epidemiologia, Agencia de Salud Pública de Barcelona, Barcelona, Spain; Lee Kong Chian School of Medicine, SINGAPORE

## Abstract

**Objective:**

To determine the case-fatality rate (CFR) at the end of the intensive phase of tuberculosis (TB) treatment, and factors associated with fatality.

**Methods:**

TB patients diagnosed between 2006 and 2013 were followed-up during treatment. We computed the CFR at the end of the intensive phase of TB treatment, and the incidence of death per 100 person-days (pd) of follow-up. We performed survival analysis using the Kaplan-Meier method and Cox regression, and calculate *hazard ratios* (HR) and 95% confidence intervals (CI).

**Results:**

A total of 5,182 patients were included, of whom 180 (3.5%) died; 87 of these deaths (48.3%) occurred during the intensive phase of treatment, with a CFR of 1.7%. The incidence of death was 0.028/100 pd. The following factors were associated with death during the intensive phase: being >50 years (HR = 36.9;CI:4.8–283.4); being retired (HR = 2.4;CI:1.1–5.1); having visited the emergency department (HR = 3.1;CI:1.2–7.7); HIV infection (HR = 3.4;CI:1.6–7.2); initial standard treatment with 3 drugs (HR = 2.0;CI:1.2–3.3) or non-standard treatments (HR = 2.68;CI:1.36–5.25); comprehension difficulties (HR = 2.8;CI:1.3–6.1); and smear-positive sputum (HR = 2.3-CI:1.0–4.8).

**Conclusion:**

There is a non-negligible CFR during the intensive phase of TB, whose reduction should be prioritised. The CFR could be a useful indicator for evaluating TB programs.

## Introduction

Tuberculosis (TB) is a curable disease and is considered to be a cause of unnecessarily premature and avoidable deaths [1] that should be eradicated as a cause of death [2]. While global TB mortality is estimated to have decreased by 45% between 1990 and 2013, it was still the cause of 1.5 million deaths in 2014, more than the number caused by HIV (1.2 million) [3]. Moreover, studies show that the death rate among TB cases is underestimated [4], and TB is often not the cause of death but rather a contributing factor [2]. Furthermore, mortality among TB patients varies considerably between countries. In Europe it was ~7% in 2012 [5], varying from 12% in Slovakia to 5% in The Netherlands [6] and United Kingdom [7]. Other studies indicate mortality of 6.6% in the United States and Canada [8], 5.5% in Shangai [9], 12% in Rwanda [10], 18% in the Chiapas region of Mexico [11], 19.7% in Taiwan [12], and 26.6% in Pakistan [13]. There is little data on mortality and survival among TB patients, and a recently European study on the topic [6] indicated that no information was available for Spain.

It is widely known that an important percentage of deaths among TB patients occur during the early phases of anti-TB therapy. However, to date few studies have investigated the case fatality rate (CFR) during the intensive phase of treatment [14–15]. The identification of the characteristics of patients who die prematurely during Tb treatment could help identify vulnerable populations and help to target them in future public health interventions. Thus, the main objective of the present study was to determine the CFR and its associated factors during the intensive phase of TB treatment in a large cohort of patients.

## Materials and Methods

Design: A population-based prospective cohort study.

Study subjects: Patients diagnosed with TB between 1 January 2006 and 31 December 2013 in one of 61 Spanish hospitals by a member of the Working Group of Integrated Programme of Tuberculosis Research.

### Identification and follow-up of cases

Inclusion criteria: 1) Positive smear-test, or negative but with culture positive for *Mycobacterium tuberculosis*; or, in cases of extrapulmonary TB, demonstration of caseating granulomas in histology. 2) Patients with suspected TB (based on clinical, radiological, epidemiological or laboratory results). 3) Age >18 years old; 4) Informed consent given.

Cases were followed up according to the assessment schedule provided in Table 1. The cause of death was decided and documented by the managing physician.

**Table 1:** Evaluation schedule of cases diagnosed of tuberculosis.

	Visit 1	Visit 2	Visit 3	Visit 4
	Diagnosis	2 Months	6 Months	9,12,18 Months (Optional)
**Inclusion/Exclusion criteria**	x			
***Socio-demographic data***	x			
***Smoking/alcohol habits***	x			
**Anthropometrics**	x	x	x	x
**Clinical history**	x			
**Diagnostic methods**	x			
***Drug treatment***	x	x	x	x
**Clinical response**		x	x	x
**Treatment adherence**		x	x	x
**Sputum sample collection**	x	x	x	x
**Drug susceptibility testing**	x			
**Treatment outcome**			x	x

All data were via a standardized online questionnaire, and were stored in an electronic case report; only the doctor in charge of the patient had access to the patient’s data, via a username and password specified for each collaborating study investigator. We created a database, which was the responsibility of our project manager.

### Variables

The variables studied were: sex; age; country of origin; employment status; living arrangements; comprehension difficulties; health center that made the diagnosis and followed up the case; history of consumption of tobacco, alcohol and injected drugs; HIV infection; previous TB treatment; TB localisation; microbiology; Chest-X-Ray; drug resistance; treatment regime; initial and final dates of intensive treatment phase; date of end of treatment; treatment outcome (cured, treatment completed, therapeutic failure, moved away/transferred, lost to follow-up, prolongation of TB treatment, and death due to TB or other cause with date of death).

### Statistical Analysis

We carried out a descriptive study of qualitative and quantitative variables to characterize the study population. We computed the frequency distributions of the qualitative variables, and compared proportions using the χ^2^ test, or the two-tailed Fisher test when expected values were >5.

We computed the incidence of death during the intensive phase of TB treatment per 100 person-days (pd) of follow-up. The duration of follow-up was defined as the interval from the date when treatment began until the end of the intensive phase of anti-tuberculosis treatment (2 months). The CFR was calculated as the percentage of deaths during the intensive phase among patients who started treatment.

In the bivariate analysis, the probability of death was determined using the Kaplan-Meier method, and survival curves were compared using the Log-Rank test. We fit bivariate and multivariate Cox proportional hazards models. We used the *backward stepwise* method of selection for the Cox multivariate model, including associated factors (p<0.05) from the bivariate level. All variables without collinearity were included in the final model and interaction between covariates was assessed.

We calculated measures of association (hazard ratios, HR) and their 95% confidence intervals (CI), and considered p-values<0.05 as statistically significant. Proportionality of hazards was assessed using Schoenfeld residuals. All analyses were conducted using version 3.1.1 of the R statistical package.

### Ethical aspects

In accordance with the International Directory for Ethical Revision of Epidemiological Studies (Council for the International Organizations of Medical Sciences—CIOMS, Geneva, 1991), and the Spanish Epidemiology Society recommendations on ethical aspects of epidemiological research, this study was approved by the Teknon Institutional Review Board (IRB). This institution acted as the reference IRB for all participant centers during 2011, when the grant was requested. According to Spanish law (Orden SAS/3740/2009), this type of study only requires the approval of a reference IRB, and were contacted prior to data collection.

All participants gave written informed consent for their clinical records to be used in this study. All records with patient identification information were handled in a confidential manner according to the ethical principles of the Helsinki Declaration and in accordance with the Data Protection Spanish Law 15/1999.

## Results

A total of 5,182 TB cases were studied. Their median age was 39 years, 62% were males, 31.6% were foreign-born, 4.2% were HIV-infected, 49.4% were attended at hospital emergency departments, 77.4% had pulmonary TB, 32.8% presented X-ray cavitation, 6.9% were resistant at least to one drug, and 6.7% had difficulty understanding the disease and/or the treatment (Table 2).

**Table 2:** Characteristics of 5,182 patients with tuberculosis, and factors related with the probability of death during the intensive treatment phase. Bivariate and multivariate analysis.

	Total (%)	Deaths (%)	Bivariate analysis	p-value	Multivariate analysis	p- value
	N = 5182	N = 87 (1.7)	HR (95% CI)		HR (95% CI)	
**Sex:**						
Female	1863 (35.9)	19 (1.0)	Ref.	Ref.		
Male	3208 (62.0)	66 (2.1)	2.03 [1.22;3.38]	**0.007**		
NC	111 (2.1)	2 (1.8)	1.78 [0.42;7.65]	0.437		
**Age:**						
18–30	1439 (27.8)	1 (0.1)	Ref.	Ref.	Ref.	Ref.
31–50	2103(40.6))	16 (0.8)	11.0 [1.46;82.8]	**0.020**	8.96 [1.18;67.99]	**0.033**
>50	1525 (29.4)	70 (4.6)	67.4 [9.37;485.0]	**<0.001**	36.92[4.81;283.39]	**<0.001**
Unknown	115 (2.2)	0 (0)	0.00 [0.00;inf]	0.995	0.00 [0.00;inf]	0.995
**Country of origin:**						
Other	1637 (31.6)	8 (0.5)	Ref.	Ref.		
Spain	3545 (68.4)	79 (2.2)	4.59 [2.22;9.50]	**<0.001**		
**Employment status:**						
Active	2750 (53.0)	11 (0.4)	Ref.	Ref.	Ref.	Ref.
Retired	885 (17.1)	55 (6.2)	16.0 [8.37;30.5]	**<0.001**	2.40 [1.11;5.14]	**0.024**
Unemployed	1247 (24.1)	15 (1.2)	3.03 [1.39;6.59]	**0.005**	1.99 [0.88;4.50]	0.097
Unable to work	107 (2.1)	1 (0.9)	2.33 [0.30;18.0]	0.418	0.70 [0.09;5.61]	0.744
Unknown	193 (3.7)	5 (2.6)	6.59 [2.29;19.0]	**<0.001**	0.97 [0.31;3.00]	0.970
**Living arrangements:**						
Group	555 (10.8)	2 (0.4)	Ref.	Ref.		
Alone	477 (9.2)	12 (2.5)	7.03 [1.57;31.4]	**0.011**		
Homeless	75 (1.4)	4 (5.3)	15.1 [2.77;82.5]	**0.002**		
Confined	63 (1.2)	2 (3.2)	8.93 [1.26;63.4]	**0.029**		
Family	3877 (74.8)	63 (1.6)	4.52 [1.11;18.5]	**0.036**		
Unknown	135 (2.6)	4 (3.0)	8.40 [1.54;45.9]	**0.014**		
**Where care received:**						
Primary care	970 (18.7)	5 (0.5)	Ref.	Ref.	Ref.	Ref.
Host. emergency dept.	2558 (49.4)	56 (2.2)	4.27 [1.71;10.7]	**0.002**	3.06 [1.21;7.70]	**0.017**
Area specialist	797 (15.4)	11 (1.4)	2.68 [0.93;7.72]	**0.067**	2.35 [0.80;6.86]	0.117
Other	857 (16.5)	15 (1.8)	3.41 [1.24;9.37]	**0.018**	1.72 [0.61;4.82]	0.296
**Smoking:**						
Non-smoker	2494 (48.1)	36 (1.4)	Ref.	Ref.		
Smoker	2640 (50.9)	47 (1.8)	1.24 [0.80;1.91]	0.340		
Unknown	48 (0.9)	4 (8.3)	5.93 [2.11;16.7]	**<0.001**		
**Alcohol:**						
No	3937 (76.0)	55 (1.4)	Ref.	Ref.	Ref.	Ref.
Yes	1245 (24.0)	32 (2.6)	1.85 [1.20;2.86]	**0.006**	1.29 [0.79;2.10]	0.298
**Injecting drug user:**						
No	5120 (98.8)	83(1.6)	Ref.	Ref.		
Yes	62 (1.2)	4 (6.5)	4.08 [1.50;11.1]	**0.006**		
**HIV:**						
No	4034 (77.8)	55 (1.4)	Ref.	Ref.	Ref.	Ref.
Yes	219 (4.2)	10 (4.6)	3.38 [1.73;6.64]	**<0.001**	3.36 [1.85;7.17]	**0.001**
Unknown	929 (17.9)	22 (2.4)	1.75 [1.06;2.86]	**0.027**	1.21 [0.73;2.02]	0.452
**Prior TB treatment**						
Yes	375 (7.2)	5 (1.3)	Ref.	Ref.		
No	4807 (92.8)	82 (1.7)	1.28 [0.52;3.17]	0.588		
**Localization:**						
Pulmonary	4009 (77.4)	64 (1.6)	Ref.	Ref.		
Extrapulmonary	487 (9.4)	10 (2.1)	1.29 [0.66;2.52]	0.447		
Unknown	686 (13.2)	13 (1.9)	1.19 [0.66;2.16]	0.567		
**Microbiology:**						
Culture(-)	922 (17.8)	8(0.9)	Ref.	Ref.		
Smear (+)	2792 (53.9)	51 (1.8)	2.12 [1.00;4.46]	**0.049**	2.32 [1.04;4.76]	**0.038**
Smear(-) / Culture (+)	1468 (28.3)	28 (1.9)	2.21 [1.01;4.84]	**0.048**	1.40 [0.63;3.12]	0.401
**Chest X-ray:**						
Normal	537 (10.4)	7 (1.3)	Ref.	Ref.		
Abnormal, cavitation	1700 (32.8)	28 (1.6)	1.27 [0.55;2.90]	0.576		
Abnormal, no cavitation	2785 (53.7)	49 (1.8)	1.35 [0.61;2.98]	0.457		
Unknown	160 (3.1)	3 (1.9)	1.45 [0.37;5.59]	0.592		
**Drug resistence:**						
No	4826 (93.1)	79 (1.6)	Ref.	Ref.		
Yes	356 (6.9)	8 (2.2)	1.38 [0.66;2.85]	0.390		
**Initial treatment:**						
4 drugs	2467 (47.6)	23 (0.9)	Ref.	Ref.	Ref.	Ref.
3 drugs	2416 (46.6)	48 (2.0)	2.15 [1.31;3.53]	**0.003**	1.96 [1.17;3.30]	**0.010**
Other	299 (5.8)	16 (5.4)	5.91 [3.12;11.2]	**<0.001**	2.68 [1.36;5.25]	**0.004**
**Patient comprehension:**						
Easy	4665 (90.0)	29 (0.6)	Ref.	Ref.	Ref.	Ref.
Difficult	346 (6.7)	8 (2.3)	3.76 [1.72;8.22]	**0.001**	2.76 [1.25;6.08]	**0.011**
Unknown	171 (3.3)	50 (29.2)	59.4 [37.6;93.9]	**<0.001**	38.34[23.49;62.5]	**<0.001**
**Distribution by period:**						
2006–2009	3034 (58.5)	39 (1.3)	Ref.	Ref.		
2010–2013	2148 (41.5)	48 (2.2)	1.76 [1.15;2.68]	**0.009**		

HR: *Hazard ratio;* CI: Confidence interval; Host. emergency dept.: Hospital emergency department; HIV: Human immunodeficiency virus; TB: tuberculosis

A total of 180 patients (3.5%) died during TB treatment, 87 (48.3%) during the intensive phase. Of these deaths, 34 (39.1%) were atributted to TB.

**Fig 1:**
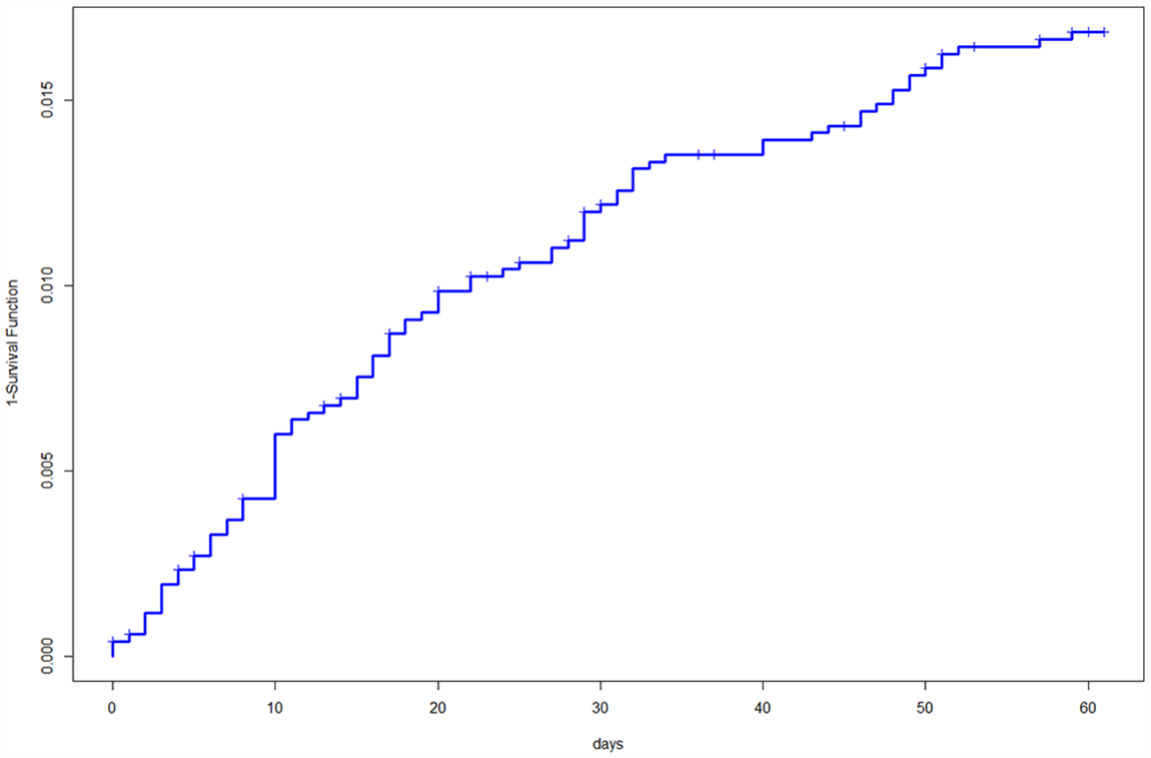
Probability of dying during the intensive treatment phase in a cohort of 5,182 tuberculosis patients.

The incidence of death during the intensive phase was 0.028/100 pd. The CFR during the initial phase was 1.7% (0.7% due to TB) and the cumulative probabilities of dying at 15, 30, 45 and 60 days were 0.8%, 1.2%, 1.4% and 1.7%, respectively (Fig 1). The intensive phase CFR during the study period as follows: 0.8%, 1.8%, 2.1%, 1.1%, 1.9%, 2.7%, 2.1% and 1.8% in 2006 to 2013, respectively. Dividing the study into two periods, 2006–2009 and 2010–2013, the CFR was higher during the second period (1.3% vs 2.2%; p = 0.009).

CFRs for various categories of the variables are shown in Table 2. The bivariate analysis showed higher mortality among men, people aged ≥ 30–50, those born in Spain, retired people, the unemployed, individuals diagnosed in hospital emergency departments or health centers that were not specialist in this area, alcoholics, injecting drug users, individuals infected with HIV, those with bacteriological confirmation, those who were initially treated with 3 drugs or other non-standard forms of treatment, patients with comprehension difficulties, and cases diagnosed during the period 2010–2013 (Table 2).

**Fig 2:**
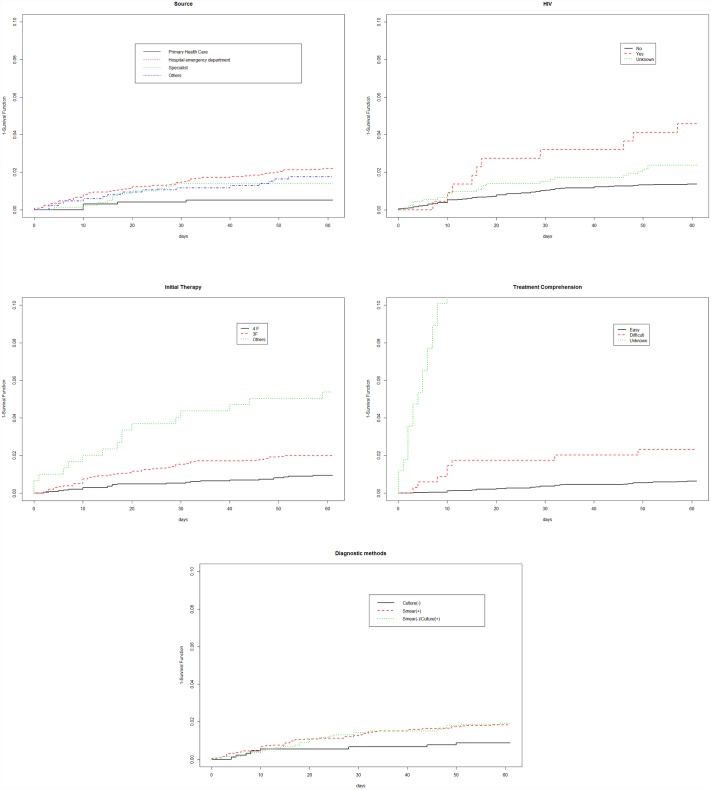
Probability of death in terms of the main factors that influence mortality during the initial treatment phase.

The Kaplan-Meier curves for the main factors that significantly affected probability of death during the intensive phase of TB treatment are shown in Fig 2.

The multivariate analysis showed that the following factors were associated with a higher probability of death during the intensive phase of TB treatment: being aged 31–50 or >50 years; being retired; having been attended at the emergency department; being HIV-infected; being sputum smear-positive; having received initial standard treatment with 3 drugs or with non-standard therapies, and having comprehension difficulties (Table 2).

## Discussion

In this study, which was conducted in a high income country, we observed a non-negligible CFR during the first two months of TB treatment, and that this was associated with factors such as age, being retired, receiving emergency care, HIV-infection, smear positivity, initial treatment with 3 drugs, non-standard therapies, and having comprehension difficulties.

While the percentage of deaths overall is lower than that observed in other European countries [5], nearly half of these deaths occured during the intensive phase of TB treatment. Moreover, 39% of deaths during this phase were caused by TB. The observed probability of death by the end of the intensive phase of TB treatment (1.7%) was lower than that reported for a study carried out in India: 2% [14], (2.6% [15] among patients treated using directly observed therapy, DOT).

Regarding factors associated with death during the intensive phase of TB treatment, the influence of age in this study was consistent with previous reports [15], with a much higher HR in individuals over 50 years of age. This may be due to comorbidities and the natural decline in immunity after age 60 years [16].

Cases diagnosed in hospital emergency departments (that are referred to a TB centre and are hospitalized for starts treatment or starts treatment at home under the control of TB area specialist) could be clinically more severe, often involving patients with few resources who contacting health services long after symptoms appear, leading to diagnostic delay and poor evolution. In addition, the higher mortality among smear-positive patients is logical since this represents more advanced forms of TB.

Some studies show that a higher CFR and poorer survival in TB patients occurs in more vulnerable population groups, such as injecting drug users, those living in poorer areas [17], those requiring intensive care [18], people with HIV infection [19], resistance to anti-TB drugs [20], a high degree of lung destruction, [21] and individuals who make inappropriate use of health services [22]. In our study however, HIV infection is the only factor associated with early death, despite the fact that mortality has fallen significantly in this group with the use of highly active antiretroviral therapy (HAART) [23].

Early death among patients treated with 3 drugs may be because of inappropriate treatment in patients with undetected drug sensitivity or resistance. In Spain the prevalence of resistances is low [24] and no diferences were observed in our study by resistances (Table 2), however the people under 3 drugs treatment had a worse survival in relation to people under 4 drugs treatment (HR = 1.96; 95% CI: 1.17–3.30). This is consistent with a study that highlighted inappropriate therapy during the intensive treatment phase as a risk factor for death [25]. Moreover, cases treated with non-standard therapies are generally patients with more complex and more severe disease (drug resistance, toxicity, intolerance, interactions or therapeutic failures).

Difficulty in comprehending the treatment regime and adherence is widely known to contribute to poor evolution and higher CFR [15]. Our study shows that this is also an independent risk factor for early death, and that the death of patients with comprehension difficulties is more likely to be due to TB than to other causes (75% vs 25%; p<0.001).

Note that mortality may be reduced more rapidly than incidence since early TB treatment would reduce both transmission and the number of deaths [26]. Thus, it would be interesting to identify modifiable risk factors associated with death, with the aim of improving clinical outcomes. One of the goals of the WHO Stop TB strategy is to reduce TB mortality [27], and DOT has been shown to be the principal action required to achieve this goal [28–30]. Other measures can also reduce this risk, such as: exhaustive follow-up of older patients [21]; HIV control [31]; early diagnosis, rapid diagnostic techniques [32]; implementation of therapies that prevent and control resistance, multidrug-resistant and extensively drug resistant TB [33,34]; and strategies that improve understanding of therapy and adherence.

The main limitation of this study is the missing data for some patients’ dates of death, which is necessary for survival analyses; however, the rate of missingness was minimal and did not affect the study’s objective because these patients died in the maintenance phase of therapy. Other limitations of this study could be the no inclussion of diagnosis delay (very difficult to objectivate) and other comorbidities not included in our report form. While it was not possible to evaluate CD4 count as a factor related to probability of dying, HIV patients with TB have access to HAART.

The strengths of this study include the large sample size and the fact that the literature review identified very few publications that studied mortality during the intensive phase of treatment.

## Conclusions

The CRF during the intensive phase of TB treatment phase is non-negligible, as almost half the deaths occurred during this initial phase of treatment. This CFR could be a good indicator for evaluating TB programs because it is available early: 2 months after starting treatment. This would allow program managers to identify a variety of factors in which to intervene (e.g. diagnostic delay, inappropriate treatment, resistance, HIV infection), thereby improving survival among TB patients.
